# Effectiveness of a digital safety plan to prevent suicidal crisis

**DOI:** 10.1192/j.eurpsy.2026.10301

**Published:** 2026-07-31

**Authors:** M. L. Barrigon, SamrtCrisis group

**Affiliations:** Psychaitry and Mental Health, Hospital General Universitario Gregorio Marañon, Madrid, Spain

## Abstract

**Background:**

Suicide is a leading cause of premature mortality worldwide and a major public health concern, particularly among individuals recently discharged from emergency departments after a suicidal crisis. Safety planning is currently considered a **gold**-standard brief intervention to mitigate short-term suicide risk, yet its implementation and use in real-world settings face important barriers related to accessibility and continuity of use outside clinical encounters. Digitalization offers a promising opportunity to extend and enhance safety planning by delivering just-in-time, scalable, and user-centered preventive tools that patients can activate during suicidal crises in their daily lives.

**Objective:**

To assess whether activating a digital safety plan integrated into the MeMind app reduces subsequent emergency department visits for suicidal ideation or attempts after an index suicide-related visit; this study is part of the Smartcrisis project for suicide behavior prevention conducted in Madrid, Spain.

**Methods:**

A one-year follow-up was conducted with patients presenting to the emergency department with an initial suicidal behavior episode (N=78), who were provided with a mobile-based digital safety plan and instructed to activate it during future suicidal crises.

**Results:**

At one year, patients who activated the digital safety plan showed an approximately 50% lower likelihood of returning to the emergency department for suicidal behavior compared with those who did not activate it.

**Conclusions:**

Activation of digital safety plans may represent a brief, scalable, and easily accessible secondary prevention strategy for suicidal behavior in emergency care settings

**Disclosure of Interest:**

None Declared.

